# The Rubber Question

**Published:** 1873-04

**Authors:** 


*The Rubber Question.*—Onr thanks are due to Dr. S. S. White for the following information concerning the Gardner suit:

"The Supreme Court of the United States has sustained my motion to vacate the decree in the Gardner suit, because of collusion."

This will be welcome news to the dental profession throughout the country.

